# Modular reactivation of Mexico City after COVID-19 lockdown

**DOI:** 10.1186/s12889-022-13183-z

**Published:** 2022-05-13

**Authors:** Guillermo de Anda-Jáuregui, Lourdes García-García, Enrique Hernández-Lemus

**Affiliations:** 1 Computational Genomics Division, National Institute of Genomic Medicine, México City, México; 2 Center for Complexity Sciences, Universidad Nacional Autónoma de México, México City, Mexico; 3 Programa de Cátedras CONACYT, Consejo Nacional de Ciencia y Tecnología, Mexico City, Mexico; 4 Centro de Investigación Sobre Enfermedades Infecciosas, Instituto Nacional de Salud Pública, Cuernavaca, México

**Keywords:** Reactivation after lockdown, COVID-19, Network Epidemiology, Mexico City

## Abstract

**Background:**

During the COVID-19 pandemic, the slope of the epidemic curve in Mexico City has been quite unstable. Changes in human activity led to changes in epidemic activity, hampering attempts at economic and general reactivation of the city.

**Methods:**

We have predicted that where a fraction of the population above a certain threshold returns to the public space, the negative tendency of the epidemic curve will revert. Such predictions were based on modeling the reactivation of economic activity after lockdown using an epidemiological model resting upon a contact network of Mexico City derived from mobile device co-localization. We modeled scenarios with different proportions of the population returning to normalcy. Null models were built using the Jornada Nacional de Sana Distancia (the Mexican model of elective lockdown). There was a mobility reduction of 75% and no mandatory mobility restrictions.

**Results:**

We found that a new peak of cases in the epidemic curve was very likely for scenarios in which more than 5% of the population rejoined the public space. The return of more than 50% of the population synchronously will unleash a magnitude similar to the one predicted with no mitigation strategies. By evaluating the tendencies of the epidemic dynamics, the number of new cases registered, hospitalizations, and recent deaths, we consider that reactivation following only elective measures may not be optimal under this scenario.

**Conclusions:**

Given the need to resume economic activities, we suggest alternative measures that minimize unnecessary contacts among people returning to the public space. We evaluated that “encapsulating” reactivated workers (that is, using measures to reduce the number of contacts beyond their influential community in the contact network) may allow reactivation of a more significant fraction of the population without compromising the desired tendency in the epidemic curve.

**Graphical Abstract:**

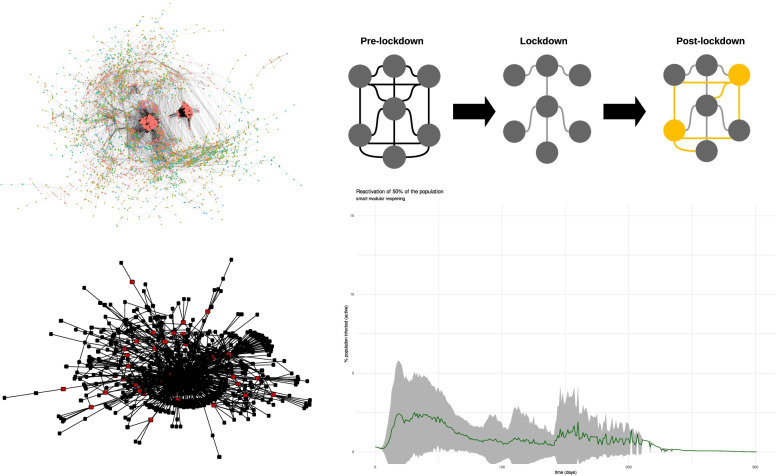

**Supplementary Information:**

The online version contains supplementary material available at 10.1186/s12889-022-13183-z.

## Background

COVID-19, caused by the novel coronavirus SARS-CoV-2, first detected in Wuhan, China, in Dec 2020, is a complex disease of infectious origins. The SARS-CoV-2 is transmitted via aerosols and respiratory droplets [1] and can unleash not only the characteristic severe acute respiratory syndrome (COVID-19) but also a series of immune and inflammatory responses that may lead to pneumonia and sepsis-induced systemic failure (SISF) [2]. We still need to understand its pathogenesis and transmission. However, current evidence shows that the spectrum of COVID-19 ranges from a mild respiratory illness to a complex, severe disease with a high mortality rate, often requiring critical care.

The enormous burden of COVID-19 has resulted from highly interconnected contact networks, causing extensive transmission of the SARS-CoV-2 virus. [3]. For this reason, it is crucial to understand the dynamics of disease spread in complex human interaction networks [4, 5].

### A primer on network epidemiology

Network Epidemiology, or Epidemics on Networks (EoN), has been defined as the study of the spread of disease and risky behaviors among populations founded on the tenets of network science [6, 7].

EoN is then concerned with the modelization of disease spread and contagion and diffusion processes happening amidst social (especially public) spaces in living systems. This approach has mainly been applied to human populations but can also be adapted to deal with animal plagues and epidemics on livestock. EoN aims to build realistic, mechanistic models to explain the spread of human disease by considering individual and collective mobility and population and meta-population features influencing the contact between individuals leading to contagion events.

These models may be used to forecast the spread of infectious diseases or risk behaviors. As in clinical and social epidemiology, these models allow us to determine risks, adopt suitable containment strategies and assess the effectiveness of targeted interventions [7].

The default setting of EoN considers the individuals in a population as nodes in a network. The interactions connecting the individuals (i.e., the links) are seen as contact events that lead to the propagation of contagious agents (pathogens). The network structure influences the pandemic's dynamic behavior and may offer clues about the mitigation and containment strategies required to contain disease spread.

The challenges posed by COVID-19 will undoubtedly impact the short, medium, and long term. The appearance of variants of concern has caused new waves of infection. Public health experts predict that we will be experiencing the emergence of new variants or new pathogens in future years and that a complete return to pre-pandemic behaviors is improbable as new pathogens will emerge. Our study helps provide preventive measures in a large metropolis like Mexico City. In this study, we compared the impact of reactivation over magnitude, timing, and the likelihood of new COVID-19 peaks according to two mobilization scenarios (modular networking *versus* constraint-free reactivation) in Mexico City and its surrounding metropolitan area after non-mandatory lockdown.

### Network modeling of human interactions using mobile devices

Human contact networks exhibit heterogeneous connectivity. The properties of this type of network are the object of study of Network Science [8]. Such heterogeneous connectivity patterns have consequences in the epidemiological setting, such as a high variance in the individual reproductive number and the dominance of the super-spreading events that arise due to the high degree nodes [7].

A significant challenge for EoN is the accurate representation of contact networks for a given population. We used a combination of observational and technology-based methods to reconstruct contact networks in limited, enclosed settings such as schools [9], hospitals [10], or conferences [11]. However, the challenge of reconstructing a contact network at the scale of a city is non-trivial.

Mexico City is the largest city in the country. It has a population of over 9 million people. According to the most recent census data, it is also the center of the Greater Mexico City Metropolitan Area (Zona Metropolitana del Valle de Mexico, ZMVM), which houses over 23 million people, according to the most recent census data [12]. Like many other large metropolitan areas, the population is highly heterogeneous in income, access to services, etc. An important issue is high heterogeneity in transportation time, and distances traveled, with a subset of the population involved in long daily commutes [13]. While these mobility patterns have been described, it is unclear whether they lead to close contacts through which the SARS-CoV-2 virus can spread (see supplementary file 1).A contact network for Mexico City was recently reconstructed using anonymized mobile device locations throughout a single day [14]. This network was released as open data [15].

To run outbreak simulations effectively, we scaled down the extensive contact network for Mexico City using a methodology based on the original network's stochastic block model (SBM) structure [16, 17]. Briefly, we obtained the SBM structure of the most significant connected component of the contact network of Mexico City and scaled the size of each block to 1/10th of the original. Then, we generated a new network using the actual SBM edge probability on the scaled-down blocks. This new network captures the original topology in terms of degree distribution and clustering coefficients, which is necessary for the results of an EoN dynamics to represent the larger one [7]. These network operations were performed using the graph-tool package [18].

## Methods

### Model fundamentals and assumptions

In this study, we worked under the following set of assumptions:

1. The disease in Mexico City is transmitted over a heterogeneous network reflecting urban systems' highly hierarchical and modular structure.
2. Epidemic dynamics are guided, in part, by factors intrinsic to the virus, which exhibit stochastic behavior as a product of inter-individual biological heterogeneity [19].
3. Given assumptions 1 and 2, the distribution of the number of contagions induced by each infected individual exhibits a long tail, giving rise to specific nodes acting as contagion hubs, leading to super-spreading events.

Under these assumptions, we used the previously described contact network for Mexico City for our modeling purposes. A node in this contact network represents an inhabitant of Mexico City. A link represents a close-range physical interaction between people; considering the resolution reported in the original manuscript (less than 2 m), these contacts can potentially transmit the infectious agent.

Once we had a reliable model for the human contact network structure of Mexico City, we were able to consider epidemic processes (namely, COVID-19 pandemic) happening on top of that network structure. The following subsection is devoted to this.

### Epidemiological simulation

We performed simulations of the epidemic dynamics by using the Epidemics on Network package (EoN) [20] to simulate possible trajectories in different economic reactivation scenarios. To do so, we used a stochastic Susceptible-Exposed-Infected-Recovered (SEIR) model on the Mexico City contact network.

The EoN package uses an implementation of the Gillespie algorithm to simulate disease spreading over the contact network. In this regard, the Markovian nature of the Gillespie algorithm implies individual disease transmission events between two nodes in the network: necessarily, one infected and one susceptible. Each of these pairwise interactions has a (“per-edge”) transmission probability, derived from treating each edge as a Poisson process with a *transmission rate* **tau(ij).**

At each time step, a set of *at risk* nodes is identified, which contains all susceptible nodes which are neighbors to at least one infected node. The *infection rate* of each at risk node is the sum of all pairwise transmission rates, A system-wide *total infection rate* is then defined as the sum of all at risk nodes’ infection rates.

The “epidemic growth” on the stochastic simulations proceeds by from these at risk nodes with a probability defined as the individual infection rate over the total infection rate. The sampled nodes then move to the *exposed* state and advance to the *infected* state at an incubation rate **ypsilon(i).**

Then, each infected node may progress to the recovered compartment at a recovery rate **gamma(i).** The simulation continues until it reaches the maximum simulation time.

### Model parametrization

To capture the inter-individual variability in terms of incubation and recovery times, we used the parameters reported for the official Mexico City’s government model [21] (see Supplementary Material 1) as base values to define per node parameters as follows:

Incubation rate and recovery rates: for each node i, ypsilon(i) and gamma(i) are sampled from a uniform distribution [½*BaseValue, 2*BaseValue].

Transmission rate: Our base transmission rate was derived from the definition of R0 = TransmissionRate(average)/RecoveryRate.To capture the adoption of general hygiene measures such as widespread use of face masks, we decided to calculate the transmission rate for our model using the average reproduction number (Rt) for June, calculated as described in [22]. We use this average transmission rate ast the mean of a normal distribution, to account for certain interactions being more conducive to disease transmission. Values for tau(ij) are sampled from this distribution.

Infected and recovered nodes at the start of the simulation: We considered the percentage of population already infected and recovered at the start of the simulation, using official figures from the federal government [23]. To address the issue of potential subreporting, we used an underreporting estimation based on delay-adjusted case fatality ratio [24] which led us to a 9-times correction factor for both recovered and active cases at the start of the simulation; that is, about 2.8% of the city’s population already recovered, and about 1.3% active infections.

### Risk assessment of reactivation schemes

Mexico's initial response to the COVID-19 emergency was a non-mandatory lockdown known as Jornada Nacional de Sana Distancia (JNSD; literally: “Healthy Distance National Period”). During this period, non-essential economic activities were limited.

Based on official figures [25], there was a 75% mobility reduction within Mexico City. We represent the effect of such lockdown in network terms by taking the original CDMX network and randomly removing 75% of its edge set. This network was referred to as the JNSD network.

**Fig. 1:**
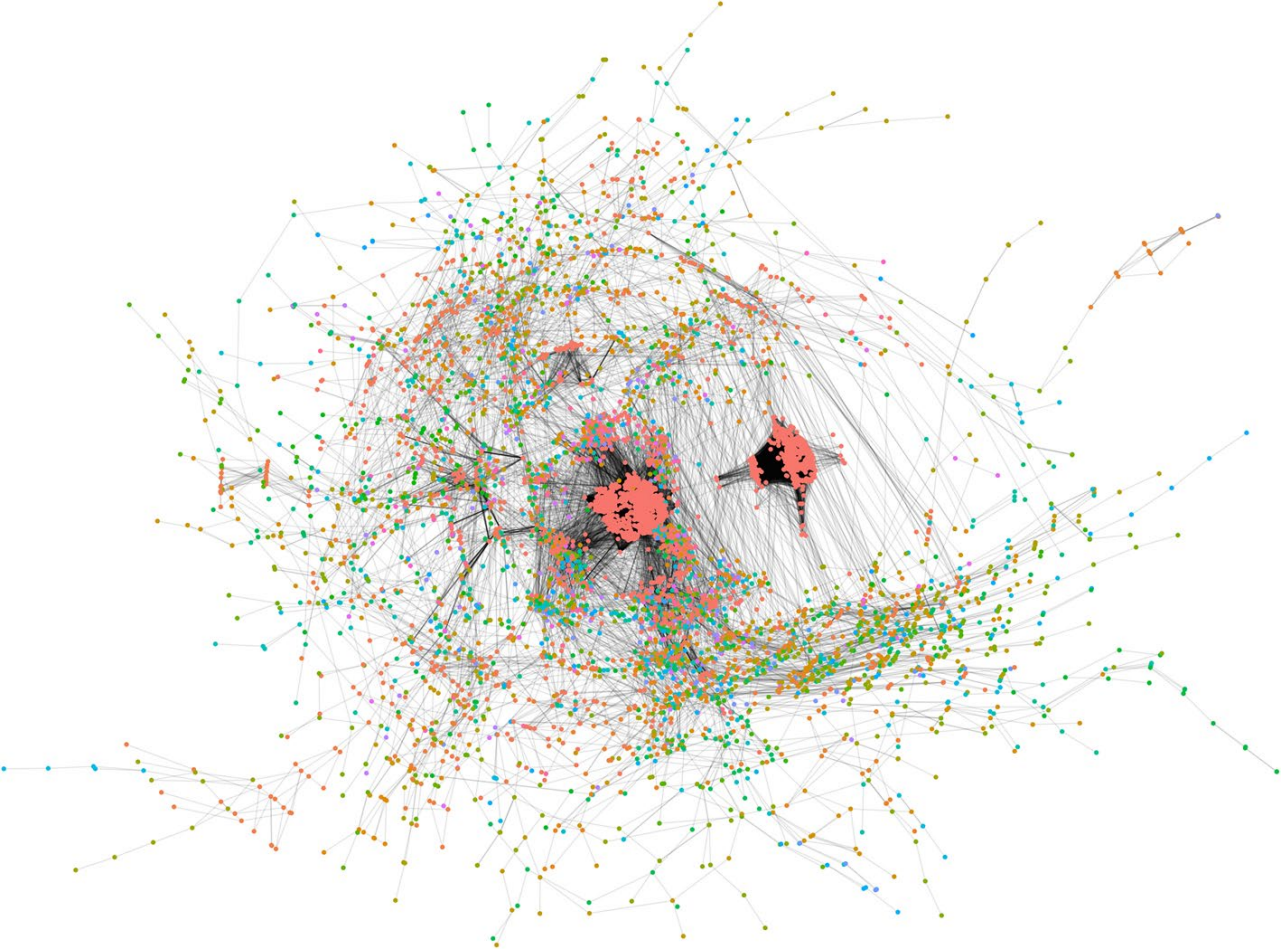
Schematic representation of the changes in contact networks induced by lockdowns and economic reactivation. In this figure, we show a simplified contact network, in which nodes represent people, and links show contacts between them

Any economic reactivation following the lockdown period implies that part of the city's population will return to regular work activities. Mobilization will lead to an increase in contact between people returning to the public space. In network terms, this is akin to the reemergence of links adjacent to the nodes representing increased interactions between people returning to the public space; for an schematic representation, see Fig. 1.

The pre-lockdown network has all the (empirically) occurring connections between people in the non-emergency state. During lockdown, many connections are lost; although some of them that are not subject to restrictions (ie. family, essential workers) remain, leading to a sparser network during the lockdown. During the economic reactivation, some of the pre-lockdown connections will be reactivated (highlighted in yellow) as people return to the public space; in addition to the connections that prevailed during the lockdown (grey), giving way to a new post-lockdown network.

With this in mind, we represented different economic reactivation scenarios by reconnecting nodes removed in the JNSD network and simulating epidemic dynamics with the parameters mentioned above. For every system analyzed, we ran 100 iterations of EoN dynamics and evaluated the behavior in terms of:

1. Whether a peak in the number of active infections occurs is measured as the number of simulations where at least one day with more infected nodes than day 0 (with a tolerance of 0.1%).
2. Percentage of the infected population at the peak: measured as the average magnitude of the peak for all simulations.
3. Peak time: measured as the average peak time for all simulations.

We present the epidemic risk evaluation under different reactivation scenarios with these ideas in mind.

The following pseudocode describes how each of this magnitudes is calculated:

### For each ensemble of simulations S:

#### For each simulation s:

${I}_{max}=max\left(I\right)$ # maximum value of I (infected percentage) at any time in s.

${t}_{max}=$ t($max\left(I\right)$) # timepoint in which I equals max(I).

$Peak=Trueiff{I}_{max}>I\left(t=0\right)+0.1$# whether the dynamics leads to an actual peak, or just decays from the initial infected nodes

$$AverageInfectionPeakMagnitude\left(S\right)=mean\left({I}_{max}\left(s\right)\right)$$

$$Averagepeaktime\left(S\right)=mean\left({t}_{max}\left(s\right)\right)$$

$$rePeaklikelihood\left(S\right)=mean\left(rePeak\left(s\right)\right)$$

### Minimum and maximum contact scenarios—JNSD and CDMX networks

The minimum level happened during the lockdown and is represented by the JNSD network. On the other side of the spectrum, the maximum level of communication is represented by the CDMX network, which captures the usual contact patterns of the city without the constraints induced by the pandemic.

### Scenarios of constraint-free reactivation

As we previously mentioned, economic reactivation involves reintegrating a fraction of the population back into the public space. This renewed activity can be represented as a reconnection of contacts removed during the lockdown.

Without any constraint and no additional information, we assumed that the workforce is evenly distributed within the population represented in the contact network [22]. Therefore, the reactivation of any fraction of the people will be akin to randomly sampling the contact network nodes and reconnecting the links lost during the lockdown. Our models were based on conservative assumptions due to scarce sociodemographic and spatial information. We evaluated this scenario using the following algorithm for different population fractions ranging from 5 to 50%.

For each fraction F of reactivated population

1. We selected F nodes of the Mexico City contact network
2. We considered that all links adjacent to other nodes were reactivated without restrictions.
3. We considered that the rest of the nodes in F (those with no correspondence to links in F) were the ones in the network as modeled under lockdown conditions as given by the JNSD network.

We used the resulting network to run an EoN dynamic using the parameters described previously.

### Scenarios of modular reactivation

An alternative approach to economic reactivation consists of leveraging network properties to impose limits on the epidemic dynamic. The concept of modularity in complex networks [24] is loosely defined as a set of nodes (individuals) with a higher number of connections among members of the group than with other nodes of the network, i.e., there are more connections within a module than between modules. An essential property of modular networks is that dynamic phenomena (such as a random walk or pathogen propagation) remain inside a module longer before spreading outside the module.

Instead of reactivating nodes spread throughout the network in our proposed modular reactivation, whole modules are reactivated until the desired fraction of the population is reactivated. All edges adjacent to the reactivated nodes are reconnected, including links within a given module and a smaller subset of connections beyond the module's boundaries.

**Fig. 2:**
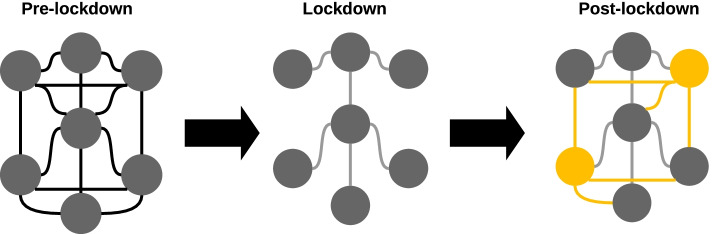
Modular interconnections and contact structure. Left: constraint-free reactivation; reactivated nodes are randomly selected from the network. Right: modular reactivation; reactivated nodes all belong to selected modules. Nodes to be reactivated are highlighted in red; the rest are black

Figure 2 illustrates and contrasts a constraint-free and modular reactivation strategy.

In the top panel, the contacts of the red nodes may include job and non-job related contacts with similar probabilities. This way, by being randomly distributed in the network, contacts are reactivated both with other essential workers (red) and with the rest of the population (black nodes), which in principle must remain under confinement (as during the lockdown).

The lower panel depicts a scenario in which red nodes form a module; hence, it is more likely to connect to other essential workers (red nodes). In case of infection within the module, the general population is, to an extent, shielded since the outbreak has a greater probability of keeping spreading within the module. Hence the attack will become contained more easily.

### Leveraging inter-module connectivity to optimize modular reactivation

**Fig. 3:**
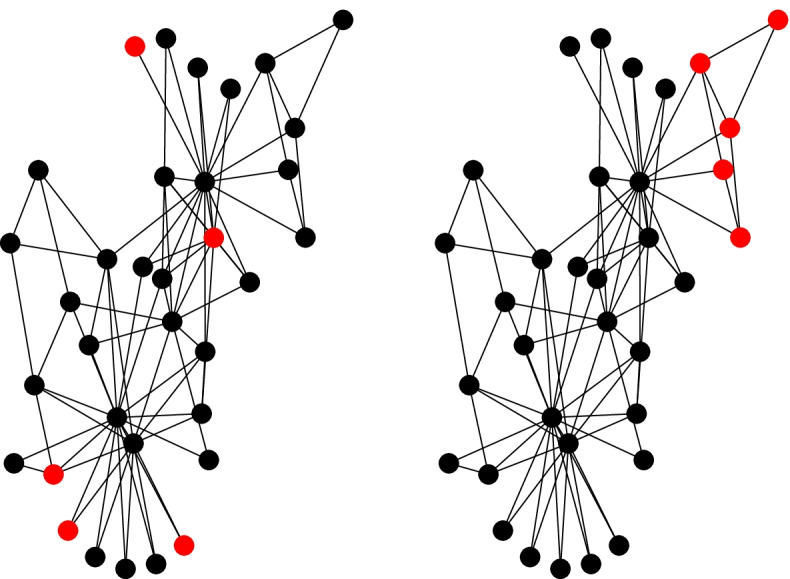
Modular projection of the CDMX contact network. These networks are aggregates of the original contact network; the nodes in these networks represent a full module in the original contact network; these modules are connected to other modules if there are connections between their member nodes in the original contact network.If we consider the number of nodes that belongs to each module, each of these modules contains a fraction of the original nodes in the network (that is, a fraction of the population of the network). In these figures, we select roughly 20% of the contact network’s population using two different strategies: in the top panel, we select (highlighted in red) the six largest modules; whereas in the bottom panel, we select 33 modules with a smaller fraction of the population, such that the sum of these is again roughly 20% of the total contact network’s population.

While modular reactivation can be accomplished by arbitrarily activating modules, the interconnection between modules can also further refine the modular reactivation. Dynamic processes will remain within a modular structure; therefore, reactivating smaller, topologically distant modules in the network will further encapsulate the epidemic phenomena and minimize its spread. See Fig. 3 for a visual representation of such strategy.

### A note on vaccination

Our current work does not consider the effect of a vaccination campaign on the evolution of the COVID-19 epidemic in Mexico City. We have decided to exclude this from our simulations based on the following considerations: 1) while there are reports that vaccines reduce SARS-CoV-2 transmission [26, 27], the initial notion was that they would provide protection, but not necessarily reduce the number of infected individuals; 2) while Mexico has an ongoing vaccination program, it has not reached the percentages of vaccination seen in other, more developed countries; 3) we consider that our model, while implemented with COVID-19 in mind, could be of use for other pandemic responses in the future, in which non-pharmaceutical interventions may again be the only available option, particularly for underdeveloped nations.

## Results

This section will present the effects of several reactivation interventions on the trajectory of the COVID-19 epidemic in Mexico City. We report epidemic curves as a percentage of the population to avoid ecological biases.

### Constraint-free reactivation quickly approaches the behavior of a full reactivation

**Fig. 4:**
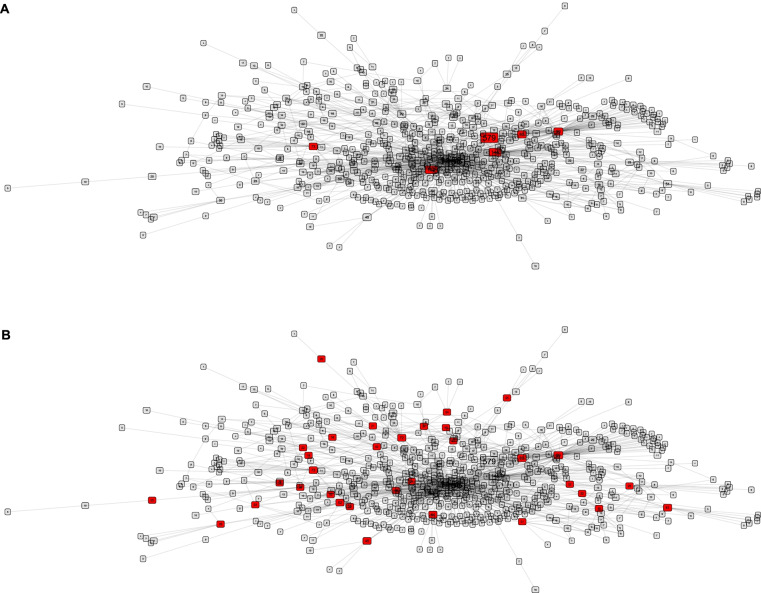
Ensemble visualization of epidemic simulations on networks. The colored line is traced to the average infected population percentage for each time point. The shadowed area represents ± 1 standard deviation. Panel A is from the simulation using the JNSD network, meaning a complete lockdown; panels B through G represent network dynamics with reactivation of 5% through 50% of the population, sampled without constraints from the network. Panel H represents the dynamics of a fully reactivated network

One initial, non-surprising result is that unconstrained reactivation of increasing fractions of the population, with no use of knowledge about the human contact network structure of Mexico City, rapidly approaches the epidemic conditions of full reactivation (Fig. 4).

Table 1 summarizes the results of the simulations under different scenarios; as the fraction of the population returning to the open public spaces increases, distribution approaches full reactivation (high peaks, longer growth curves, and higher likelihood of outbreaks).

**Table 1:** Summary statistics for constraint-free reactivation strategies

Reactivation scenario	Average Infection Peak Magnitude (%population)	Average Peak time (days)	Peak likelihood
0%	2.560421	5.43640	31%
5%	3.085227	4.66820	34%
10%	6.983370	12.70559	77%
15%	9.493764	15.40302	97%
20%	9.570953	14.99723	97%
25%	9.802384	15.31128	97%
50%	11.986835	18.73304	100%
100%	12.282289	18.42075	100%

**Fig. 5:**
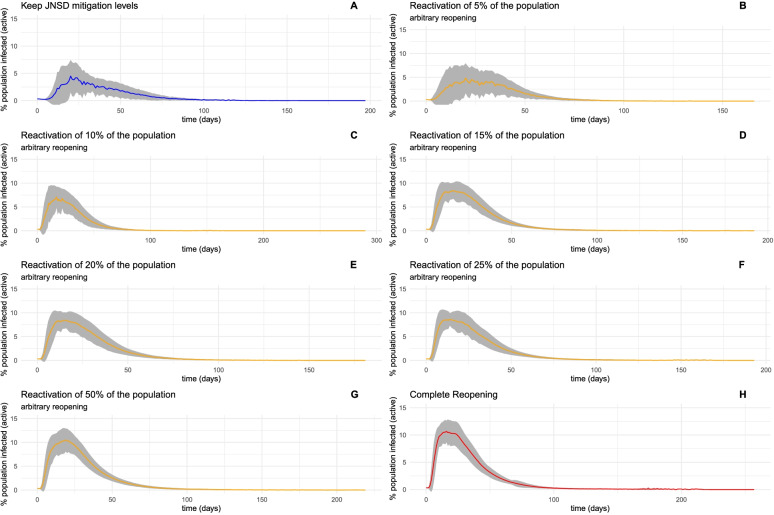
A network with 7216 vertices and 86,775 edges, representative of the close contact dynamics in Mexico City. The color of each node represents its “community,” a module of nodes that are more closely connected. We call this the CDMX network

Reactivation strategies in a randomly distributed population allow only a tiny fraction of the people to be reactivated without a high risk of resurgence of cases. We observe that any fraction beyond 5% of the population rejoining the public space leads to an increased risk of a new wave of infections. The system is quite fragile; The shadowed areas in Fig. 5, representing all the simulated trajectories, becomes narrower, and also sharper around the peak area, as random reactivation increases – indicative of less variability in the possible trajectories. Furthermore, a reactivation of 50% of the population is virtually indistinguishable from a full reactivation in terms of the magnitude, timing, and likelihood of a new peak.

### Modular reactivation is better tolerated in terms of peak magnitude and time.

**Fig. 6:**
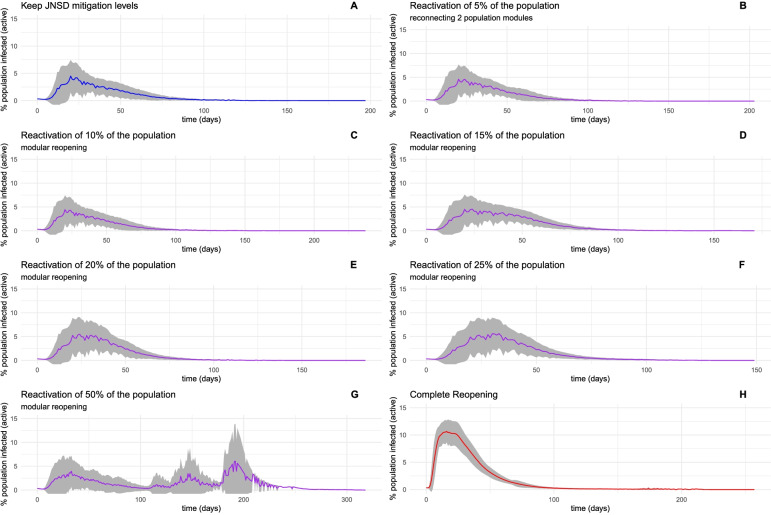
Ensemble visualization of epidemic simulations on networks. The colored line is traced to the average infected population percentage for each time point. The shadowed area represents ± 1 standard deviation. Panel A is from the simulation using the JNSD network, meaning a total lockdown; panels B through G represent dynamics on networks with reactivation of 5% through 50% of the population, achieved through the reactivation of modules in the contact network. Panel H represents the dynamics of a fully reactivated network

A second, less dramatic scenario occurs whenever we make use of some knowledge about the human contact network structure of Mexico City, namely its modular character. The height and duration of the peaks, as shown in Fig. 6, are diminished (for similar percentages of returning population) concerning the epidemic curves in the unconstrained case (Fig. 6).

A glance at the summary statistics presented in Table 2 compared to Table 1 also reveals that this is the case.

**Table 2:** Summary statistics of modular reactivation. ^∗^ Exhibits more than one peak the first peak is described

Reactivation scenario	Average Infection Peak Magnitude (%population)	Average Peak time (days)	Peak likelihood
0%	2.560421	5.43640	31%
5%	2.599640	5.51050	31%
10%	2.560560	5.40828	31%
15%	2.589800	5.52702	31%
20%	3.046286	7.43976	31%
25%	3.047949	7.72372	31%
50%	2.720205 *	11.197762	27%
100%	12.282289	18.42075	100%

Economic reactivation strategies using the modular structure of contact networks limit the spread of the infectious agent. We can observe that up to 25% of the population reactivation can be achieved with a slight deviation from the complete lockdown in magnitude, timing, and the likelihood of a new peak. In the case of a 50% modular reactivation, subsequent peaks are likely to emerge. However, it is essential to note that the magnitude of these peaks is less than that observed in a complete reactivation scenario.

Modular reactivation is thus a better alternative in allowing the incorporation of higher percentages of the population without the risk of massive outbreaks. However, as we can see, later on, there are still better alternatives nurtured by the use of deep knowledge of the topological parameters of the human contact network of Mexico City.

### Topology-guided smart selection further mitigates epidemic dynamics

**Fig. 7:**
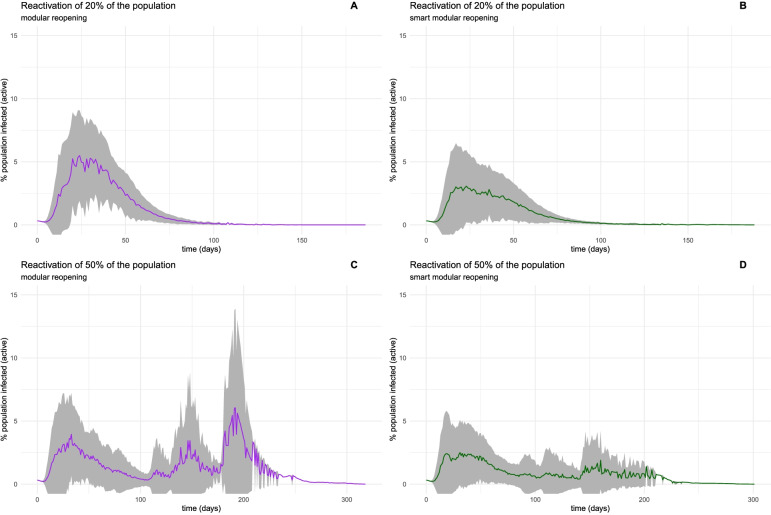
Ensemble visualization of epidemic simulations on networks. The colored line is traced to the average infected population percentage for each time point. The shadowed area represents ± one standard deviation. Panel A and B represent the dynamics with 20% population reactivation using a modular strategy. For panel A, reactivation is achieved by reactivating six modules. In contrast, the same population fraction was spread in 33 modules for panel B. Panels C and D show the same distinction, this time for 50% of the population, distributed in 164 and 247 modules, respectively. Notice that by spreading the reactivation in several smaller modules, the height of new peaks is reduced

By taking into account not only the global topological modularity of the human contact network of Mexico City but also the way the modules are connected (in the down-scaled version), we have been able to develop an optimized set of reactivation strategies, as is presented in Fig. 7 and Table 3.

**Table 3:** Summary statistics of modular reactivation. * Exhibits more than one peak; the first peak is described

Reactivation scenario	Average Infection Peak Magnitude (%population)	Average Peak time (days)	Peak likelihood
20%—6 modules	2.560421	5.43640	31%
20%—33 modules	2.560560	5.40828	31%
50%—164 modules	2.589800 *	5.527020	31% *
50%—247 modules	3.046286 *	7.439762	31% *

If the population to be reactivated is spread throughout several (more minor) modules, it is possible to control the epidemic dynamics. Even in the case of a 50% population reactivation, this "smarter" modular strategy decreases the magnitude of subsequent possible peaks.

Hence, by considering the contact network's global and local modularity structure and simulating epidemic dynamics compliant with these, we have devised an optimized progressive de-containment strategy.

## Discussion

Epidemic spread of infectious diseases occurs via chains of transmission, which are dynamic processes shaped by different human behaviors. In the case of COVID-19, the condition is spread through close human interactions [28], which can be modeled as contact networks.

Human interactions [28] can be modeled as contact networks. The contact networks of huge metropolitan urban environments present unique challenges for epidemic network modelization due to their intricate modular structure and size. Computationally efficient methods to scale down the actual (extensive) contact networks to manageable yet still descriptive sizes, capturing the relevant aspects of the modular structure of the original networks, are needed to perform epidemic dynamic models representative of the actual populations.

Using these scaled contact networks and a stochastic dynamics on networks approach, we captured the essentials of epidemic spread in Mexico City. We used the knowledge of said spreading patterns to model de-confinement scenarios to evaluate reactivation strategies after a lockdown in Mexico City. Although these networks exhibit a dynamic dimension, using a representative network as a baseline on which different lockdown and reactivation strategies can be modeled provides an efficient tool to explore different scenarios.

Since human contact networks in large urban environments tend to exhibit a modular structure, this invisible compartmentalization is one feature that shapes the transmission chains of an epidemic phenomenon. By optimizing the modular structure of the re-entrant essential worker population, we have proposed schemes that allow for a significant percentage of the population to return to public space without leading to massive outbreaks.

This work finds that economic reactivation is feasible without necessarily resulting in a new outbreak. However, for this to work, reactivation must occur within contact communities. Optimally, these modules should be small and exhibit minimal connections to limit the spread of the disease. A set of behavioral and regulatory actions are needed to constrain this contact dynamics tightly, as the alternative shows that even a small fraction of the population being arbitrarily reactivated leads to an epidemic behavior similar to that of implementing no lockdown at all.

### From the whiteboard to public policy: how to translate

Our findings might be helpful to provide feedback to public health policies [29]. In clinical fields, as is the case of medicine and nursing, a similar void is found between scientific discovery and health policy application. Several authors have suggested that establishing a program involving community participation, considering time-efficient approaches, ongoing training, and solid organizational values on evidence-based practice is necessary to disseminate scientific findings effectively. Furthermore, implementing a research discovery among government health organizations, clinical practice groups, and the general population is not immediate but is expected to proceed in stages. The decision to adopt, accept, and utilize an innovation is not an instantaneous act but more often a process [30].

The studies just referred have provided us with the following intuitions regarding modularization of the human contact structure in urban environments such as the one in Mexico City:

1. Residential proximity induces communities (people go to the same shops, etc.).
2. Non-public facing workspaces induce communities.
3. Commuting induces inter-module connections (intermixing in public transportation, recreational outings, etc.).
4. Public-facing jobs induce intermodule connections.

Based on our results, we have concluded that modular reactivation is a better alternative in allowing the incorporation of higher percentages of the population without the risk of massive outbreaks. If the population to be reactivated spreads through smaller modules, it is possible to control the epidemic dynamics. Therefore, we consider that we may formulate the following recommendations:

**Interleaved reactivation:** different, physically distant neighborhoods every day.

**Delay** the return of workers that require long commutes.

Authorities can enforce modularity by closing public transportation needed for intermodular connections; however, leaders should avoid costly penalties to the population. Effective strategies involve a significant degree of **logistic complexity** (beyond the scope of the paper) that the competent authorities must resolve.

Public health authorities must consider logistic complexities to translate the findings of this study into actionable policy. In this regard, measures that must encourage modularity reactivation are presented in Table 4.

**Table 4:** Some examples of policies to encourage/enforce modular reactivation

Policy	Government actionable	Citizenship actionable	Expected adoption
Encouraging local trade consumption habits	Suggested	Yes	Heterogeneous
Phased workplace reactivation	Suggested or mandatory	No	Suggested: Heterogeneous Mandatory: High
Turned of the workforce by residential address location	Suggested or mandatory	Yes	Suggested: Heterogeneous Mandatory: High
Partial transportation shutdown	Complete Shutdown or Turned over schedule of a fraction of public transportation spots/hubs	No	Mandatory: High if combined with complementary policies

Limitations of our model and other considerations.

Mathematical modeling implies simplification of the "real world." An aspect that we have not considered is mobility data. This factor shows a large number of long commutes within the city, which, as previously mentioned, could be a factor driving inter-module communication. Understanding these commute patterns as a function of residence could be an essential next step or the implementation of directed interventions to reduce inter-module connectivity; the main proposal of the current manuscript. This issue is discussed in the Supplementary Material. A limitation of our study is that the model we are proposing has not been tested. However, we consider it feasible based on mobility studies that have been conducted in Mexico City and surrounding metropolitan areas. Previous studies show most trips originating in municipalities on the periphery of Mexico City and the surrounding metro area have destinations in the central parts of the same municipalities. Therefore, modular networking would not significantly alter pre-pandemic mobility.

Mobility metrics have been used as an input for other COVID-19 models. The rationale is that for people to gather and have contacts, they must leave their houses for the public space. However, this is not sufficient: people may enter the public sphere but avoid interacting with other people, therefore having low risk of disease spread; importantly, in populations like Mexico City, where some populations (particularly those in low socioeconomic situations) may live in overcrowded spaces, high, long distance mobility may not even be necessary for disease spreading. In this regard, we consider that our approach, which is based on co-localization contact networks rather than mobility metrics, is better to capture the possible paths through which the virus may spread.

A central question at this stage of the pandemic is the role of vaccines in preventing transmission, which remains an open question [31], with only the mRNA-based vaccines having preliminary evidence of preventing transmission [32]. Vaccines that prevent viral transmission could be modeled as effectively disconnecting vaccinated individuals from the contact network; since these would no longer contribute to disease transmission. Therefore, vaccination modules act as hubs connecting other modules in a mesoscale sense. However, at this stage, the evidence can only confirm the protective effects of vaccines. In this regard, the effects of vaccination (reducing the risk of complications) and modularization (declining transmission) could be seen as an additive in terms of reducing public health strain (that is, reducing hospitalizations and deaths). The model described in this work is focused only on disease transmission and does not consider compartments for hospitalizations or deaths. However, future directions may assess the impact of concurrent vaccination and modularization strategies on hospital occupation and fatalities.

## Conclusions

By considering mechanisms for the reactivation of economic activities in Mexico City, we have evaluated the risk that the negative slope of the epidemic curve may be reversed by incorporating a fraction of the population into the public space.

As null models, we ran the epidemic dynamics on the contact network of Mexico City with no mobility restrictions, representing a return to the connectivity prevailing before the start of the pandemic. The second null model considered was the network corresponding to the JNSD network, a subgraph of the Mexico City contact network with just 25% of active links, capturing the effect of the reported mobility reduction. In these models, reactivated nodes are randomly distributed on the network, which is a conservative assumption due to the lack of further information on the sociodemographic and spatial distribution of the individuals involved in the activities that will be reactivated. The model we propose is a component of epidemiologic surveillance that should include timely detection of active cases and hotspots. In addition, it has the advantage of providing a rapid response to prevent further transmission and inform containment and mitigation measures.

We consider that the concept of modularity in network theory may support reactivation economic activities.

A module in a complex network is loosely defined as a set of nodes (individuals) with a higher number of connections among members of the set than with other nodes of the network, i.e., there are more connections within a module than between modules. An essential property of modular networks is that dynamic phenomena (such as a random walk or pathogen propagation) remain inside a module for longer before spreading outside the module.

By translating the findings of network-based analytics and epidemiological models into actionable public policy measures, it is possible to advance into incorporating predicted risks into the assessment portfolio for reactivation large urban conglomerates such as Mexico City after a lockdown in the face of the still ongoing Covid-19 pandemic.

## Supplementary Information

**Additional file 1.** Supplementary Material

## Data Availability

Case data: case data were obtained from the public dataset hosted by the Federal Government at: https://www.gob.mx/salud/documentos/datos-abiertos-152127 Code: all code available at https://github.com/guillermodeandajauregui/ModularReopeningCDMX
