# Supplementary material for: Modular reactivation of Mexico City after COVID-19 lockdown
Source: BMC Public Health. 2022 May 13;22:961. doi: 10.1186/s12889-022-13183-z (PMC9100316; doi:10.1186/s12889-022-13183-z)
Supplement: Supplementary file 1 — Additional file 1. Supplementary Material [file 12889_2022_13183_MOESM1_ESM.pdf]

Modular Reactivation of Mexico City After COVID-19 Lockdown
Guillermo de Anda-Jáuregui , Lourdes García-García; Enrique Hernández-Lemus

Supplementary Material:

The role of mobility in network modularity:

The question of what social phenomena drive the emergence of modules in contact networks is open. A first intuition would indicate that heterogeneous mobility patterns could lead to some groups having a higher probability of contacting each other: for instance, long-commuters being more exposed to contacts than people whose activities are predominantly performed near their residences.

In an initial analysis for this work, we reconstructed a network based on the Origin-Destination Survey (Encuesta Origen-Destino, EOD) for Mexico City (https://www.inegi.org.mx/programas/eod/2017/default.html) . This survey provides aggregated traffic volumes between regions within the Greater Mexico City Metropolitan Area. We used this information along with the population of said regions to construct a network in which the contact probability of residents of two regions is a function of the travel volumes between the two regions, adjusted for their population.

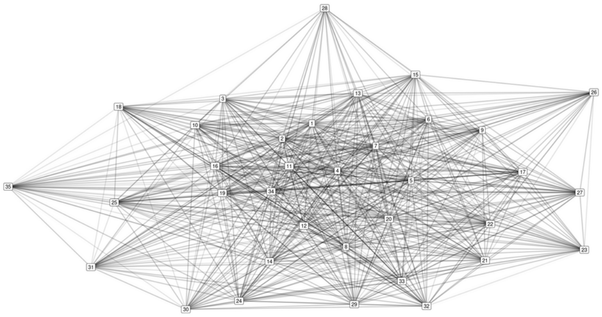

Modules of a contact network derived from EOD

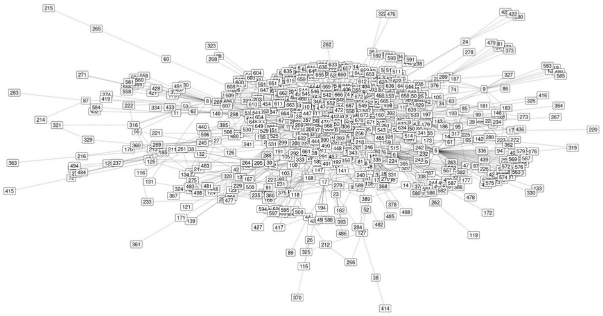

Modules of a contact network derived from empirical mobile device data.

This network exhibited a higher modularity than the network constructed using empirical data from device location (0.49 vs 0.39); yet the empirically generated network is fractured in a much larger set of modules. This discrepancy can be explained by the probabilistic nature of the first network, which

makes it less likely to find the large amount of non-connected nodes seen in the empirical network: that is, nodes that, while active, do not participate in close-contacts. This can also be explained by the data resolution: the size of the regions reported in the EOD, as well as its timescale, is such that even if two people are visiting the same region, there is no guarantee that 1) they will be visiting the same location at the same time and 2) that they will be physically close; both limitations overcome by the use of mobile device data.

Nevertheless, mobility data shows the large amount of long-commutes within the city, which as previously mentioned could be a factor driving inter-module communication. As such, understanding these commute patterns as a function of place of residence could be an important next step or the implementation of directed interventions aimed at reducing inter-module connectivity; the main proposal of the current manuscript.
